# Unique Trans-fatty Acid Profile in Children With Attention Deficit Hyperactivity Disorder

**DOI:** 10.3389/fpsyt.2021.740169

**Published:** 2021-11-05

**Authors:** Ayelet Armon-Omer, Eti Amir, Hadar Neuman, Saleh Khateeb, Itai Mizrachi, Monia Shalan, Snait Tamir, Uri Yatzkar

**Affiliations:** ^1^Research Laboratory, Ziv Medical Center, Zefat, Israel; ^2^Department of Nutrition, Ziv Medical Center, Zefat, Israel; ^3^Medical Laboratory Sciences, Zefat Academic College, Zefat, Israel; ^4^Department of Pediatrics, Ziv Medical Center, Zefat, Israel; ^5^Laboratory of Human Health and Nutrition Sciences, MIGAL Galilee Research Institute, Kiryat Shmona, Israel; ^6^Nutrition Science, Tel-Hai College, Upper Galilee, Israel; ^7^Department of Child and Adolescent Psychiatric, Ziv Medical Center, Zefat, Israel; ^8^Faculty of Medicine, Bar Ilan University, Zefat, Israel

**Keywords:** ADHD, trans-fatty acid, CGI-S, red blood cells, DHA

## Abstract

**Background:** Attention deficit hyperactivity disorder (ADHD) is the most common developmental disorder in children. Studies suggest an association between fatty acids composition and ADHD pathogenesis. We aimed to investigate whether children diagnosed with ADHD present unique fatty acid profiles in red blood cells (RBC), as compared to children without ADHD.

**Method:** We examined 60 children aged 6–14 years, out of which 32 were diagnosed with ADHD, and 28 were not. Blood was collected from all children to quantify an array of 26 fatty acids from RBC membranes. Fatty acid methyl esters were generated by acid transesterification and analyzed by gas chromatography.

**Results:** We found that children with ADHD presented unique fatty acid profiles on RBC membranes with significantly higher levels of most of the trans-fatty acids (Total trans-fatty acids 0.64 ± 0.21 vs. 0.49 ± 0.18 *p* = 0.003) and lower levels of docosahexaenoic acid (DHA), as compared to controls (4.06 ± 0.79 vs. 4.68 ± 1.37 *p* = 0.040). Additionally, total trans-fatty acids were higher in children with extremely severe clinical ADHD condition score, as compared to milder ADHD scores and to control children (0.72 ± 0.18, 0.64 ± 0.20, 0.61 ± 0.22, 0.49 ± 0.18, *p* = 0.010, accordingly).

**Conclusion:** Children with ADHD have higher levels of trans-fatty acids in RBCs, compared to children without ADHD. This study points to a possible link between trans-fatty acids and ADHD. Understanding these findings and the clinical meaning will potentially contribute to a more targeted dietary intervention.

## Introduction

Attention deficit hyperactivity disorder (ADHD) is the most common developmental disorder in school aged children (1). The prevalence rate of ADHD within Western countries is between 5 and 12%, while about 60% of these children retain ADHD symptoms into adulthood (2). Attention deficit hyperactivity disorder is defined as a persistent and pervasive pattern of inattention and/or hyperactivity-impulsivity that interferes with functioning or development and results in a negative impact on social and academic/occupational activities. Attention deficit hyperactivity disorder is a multifactorial disorder, the cause not precisely known, and its etiology is complex, involving both neuro-biological and environmental influence (3). Nutrition is one of the environmental factors possibly involved in ADHD pathogenesis (4). Currently, the exact role of nutrition is unclear, and ADHD therapy is not associated with a particular diet (5). However, ADHD symptoms have been associated and were shown to predict poorer diet quality (6). Exposure to toxins, or food additives such as dyes and preservatives was reported to promote development of ADHD (7).

It has previously been suggested that altered fatty acid composition may be associated with ADHD (8, 9). However, conflicting observations were found regarding fatty acid imbalances in ADHD in the different studies (10). Fatty acids are integral components of biological membrane phospholipids, and influence membrane fluidity, function of receptors, ion transport, and enzyme activity (11). Fatty acids can be saturated or unsaturated (containing double bonds between carbon atoms). Additionally, lower omega-3 polyunsaturated fatty acid (n-3 PUFA) levels were found to positively associate with ADHD symptom severity in children (8). One meta-analysis found that children with ADHD had lower blood levels of reliable omega-3 than typically developing children (12). Therefore, several clinical trials of n-3 PUFAs in children with ADHD have been performed, leading to controversial results. While some clinical trials showed improvement in clinical symptoms (e.g., hyperactivity/impulsivity) and cognitive performances, others did not find beneficial effects (13).

The geometry of the double bonds in lipids biosynthesized by eukaryotes is exclusively *cis* (14). Trans-fatty acids are often considered to have negative effects on human health (9, 15). They are roughly similar to saturated fats in their three-dimensional structure and may influence the melting point and the ability to fit tightly in a membrane structure. Trans-fatty acids could cause inflammation, calcification of arterial cells, and are a well-known risk factor for cardiovascular diseases (16). A study in rats showed that trans-fatty acids were incorporated into brain structures that were rich in PUFAs, suggesting this can potentially be the case in humans as well (17). Additionally, development of hyperactive behavior in rats was observed after chronic consumption of trans-fatty acids (18). However, we found only one study in humans that suggested that female adolescents with ADHD have a significantly higher intake of trans-fatty acids than those without ADHD (19). Several studies have previously linked fat consumption with ADHD. A study which reported dietary patterns in adolescents with ADHD using complete 7-day dietary records demonstrated that ADHD subjects consumed significantly higher levels of trans-fatty acids per day (3.6 ± 3.1 vs. 1.3 ± 0.7 g, *p* = 0.038) (8). Another example is an Australian study which showed that a Western diet, characterized by high intakes of saturated and trans-fat, was associated with ADHD symptoms in children (20).

One of the greatest challenges in research of different central nervous system (CNS) disorders like ADHD is that most CNS tissues are not easily accessible. Therefore, in this study we chose to measure fatty acid profiles from blood. While blood does not fully resemble what occurs within the CNS, it remains the best minimally-invasive source for measuring nutritional components (21). As fatty acids build cell membranes, the fatty acid profile of red blood cells (RBCs) reflects the long term dietary fatty acid intake averaged over the RBC lifespan (up to 120 days) of an individual. The fatty acid profiling method has been previously validated for RBC fatty acid composition. Several alterations, especially regarding levels of omega-3, have been shown in common chronic childhood diseases in Western countries such as asthma, allergy (22), depression (23), and ADHD (24).

Our aims were to test the fatty acid composition of RBC membranes in children with vs. without ADHD, and answer whether children with ADHD present a unique fatty acid profile.

## Materials and Methods

### Study Design

The study was authorized by the Ziv Helsinki Committee, Israel Ministry of Health, and registered at clinicaltrials.gov under number NCT02391428. Written informed consent was obtained from parents, and verbal assent was received from children before entering the study.

### Participants

A total of 60 volunteers between 6 to 14 years of age were recruited through the department of child and adolescent psychiatry at the Ziv Medical Center, Israel. Out of these, 32 children were diagnosed with ADHD as confirmed by a children's psychiatrist according to the criteria of Diagnostic and Statistical Manual of Mental Disorders-5 (DSM-5) (25). Presentation type of ADHD in the study included: combined presentation (*n* = 24, 75%), predominantly inattentive presentation (*n* = 7, 21.8%), and predominantly hyperactive/impulsive presentation (*n* = 2, 6.25%). In addition, 28 children without ADHD or related neuropsychiatric syndromes were recruited as a control group. Exclusion criteria for both groups included history of other psychiatric disorders and other severe chronic or autoimmune disorders. Body weight and height of subjects were measured by an electronic balanced beam scale and body mass index (BMI) was calculated.

Blood samples (3 ml) were taken from all children by venipuncture into EDTA tubes. RBCs were isolated immediately after collection by centrifugation at 2,500 g for 10 min. and then stored at −80°C for subsequent analyses. Researchers were blinded for the status of samples when performing analysis.

Fatty acid composition was analyzed by the omega-3 index methodology previously published (26). Blood fatty acid composition was assessed from separated and thawed RBCs by acid transesterification and analyzed by using gas chromatography (GC2010, Shimadzu, Duisburg, Germany) using hydrogen as a carrier gas after extraction and conversion to fatty acid methyl ester. A total of 26 fatty acids were quantified and validated by a standard mixture of fatty acids characteristic of RBCs. Results were calculated as a percentage of total identified fatty acids after response factor correction.

### ADHD Symptoms Evaluation

All children were clinically evaluated for ADHD symptoms by psychiatric examination according to the DSM-5 definitions, including the commonly used Clinical Global Impression-Severity scale (CGI-S) assessment (27). The CGI-S is a seven-point scale that assesses the overall clinical status of a subject, with scores ranging from 1 (mild) to 7 (extremely severe), relative to the clinician's past experience with patients who have the same diagnosis.

### Statistical Analysis

In order to ensure correct use of parametric statistical tests, both the ADHD and the control groups included ~30 participants as minimum size groups in this pilot study. For categorical variables, summary tables are provided giving sample size and absolute frequencies. For continuous variables, summary tables are provided giving arithmetic mean (M) and standard deviation (SD). The independent-sample *t*-test or Mann-Whitney non-parametric tests were applied for testing differences between control and ADHD groups. Kruskal-Wallis non-parametric tests were used to for testing differences according to CGI-S assessment. *p*-value of 5% or less was considered statistically significant. All data was analyzed using SPSS version 24 (SPSS Inc., Chicago, IL, USA).

## Results

### General Characteristics

The study included 32 children with ADHD, along with 28 children without ADHD, as a control group. The male-to-female ratio among ADHD participants was about 2:1 (Table 1), which is in accordance with a higher male ratio in previous epidemiological studies of ADHD (28). Gender at enrollment was comparable between the ADHD vs. control groups (66.7 vs. 50.0% males, *p* = 0.187). Mean age at enrollment was similar among the ADHD group and the control group (10.2 ± 2.0 vs. 10.3 ± 2.4 years, *p* = 0.876). The mean BMI-value of the ADHD group was 18.03 (±3.5), and mean BMI percentile was 57.86. In the control group, mean BMI-value was 15.68 (±1.9), and mean BMI percentile was 26.86 (*p* = 0.046). Both averages and percentiles were within the normal range. Children were diagnosed with ADHD according to the criteria of DSM-5 and the presentation type of ADHD included: combined presentation (*n* = 23, 71.9%), predominantly inattentive presentation (*n* = 7, 21.8%), and predominantly hyperactive/impulsive presentation (*n* = 2, 6.25%). There were no significant changes between the different groups.

**Table 1:** Demographic characteristics of the study participants.

**Variables**	**Control** **(***n*** = 28)**	**ADHD** **(***n*** = 32)**	***p*** ^*^	**ADHD CGI-S**			
				**4** **(***n*** = 10)**	**5** **(***n*** = 14)**	**6** **(***n*** = 8)**	***p*** ^**^
Gender, male (*n*, %)	14, 50.0	22, 68.8	0.187	7, 70.0	8, 57.1	7, 87.5	0.349
Methylphenidate medication (*n*, %)		13, 40.6		4, 40.0	4, 28.6	5, 62.5	0.385
Age (years, mean ± SD)	10.3 ± 2.4	10.2 ± 2.0	0.876	11.1 ± 1.8	9.9 ± 2.2	9.8 ± 2.0	0.233
Weight (Kg, mean ± SD)	31.6 ± 10.9	36.1 ± 11.5	0.156	37.9 ± 10.4	36.5 ± 11.6	30.1 ± 8.4	0.245
Height (m, mean ± SD)	1.36 ± 0.17	1.40 ± 0.13	0.429	1.44 ± 0.09	1.40 ± 0.15	1.34 ± 0.12	0.303
BMI (Kg/m^2^, mean ± SD)	15.67 ± 1.92	18.03 ± 3.45	0.046	18.1 ± 3.4	18.0 ± 2.0	16.4 ± 1.6	0.186

*ADHD, attention-deficit hyperactivity disorder; CGI-S, clinical global impression-severity scale; SD, standard deviation; BMI, body mass index*.

* *The independent-sample t-test*.

** *Kruskal-Wallis non-parametric tests were used*.

The children with ADHD either did not receive any medication (*n* = 19), or consumed only methylphenidate medication (*n* = 13, 40.6%), however no significant differences were observed in additional characteristic values between these two sub-groups (Table 2). Importantly, these two groups displayed no differences in CGI-S.

**Table 2:** Characteristics according to use of methylphenidate medication.

**Variables**	**No** **medication** **(*n* = 19)**	**Methylphenidate** **medication** **(*n* = 13)**	***P***
Gender, male (*n*, %)	11, 57.9	11, 84.6	0.141
Age (years, mean ± SD)	10.2 ± 2.3	10.3 ± 1.8	0.870
Weight (kg, mean ± SD)	36.3 ± 11.5	34.0 ± 9.5	0.558
Height (m, mean ± SD)	1.40 ± 0.14	1.40 ± 0.12	0.938
BMI (kg/m^2^, mean ± SD)	18.0 ± 2.6	17.1 ± 2.3	0.316
CGI-S (m, mean ± SD)	4.8 ± 0.7	5.1 ± 0.9	0.448

*SD, standard deviation; BMI, body mass index. CGI-S, clinical global impression-severity scale*.

### RBC Fatty Acid Profiles

We assessed whether children with ADHD present unique fatty acid profiles on RBC membranes, as compared to the non-ADHD healthy controls. We found significant differences in levels of several fatty acids when comparing the overall ADHD group with controls (Table 3). Out of the 26 measured fatty acids, 11 showed significant differences between groups; children with ADHD presented lower levels of three fatty acids, and higher levels of eight fatty acids, as compared to controls. After Bonferroni correction according to the subgroups, significance was remained only in three trans-fatty acids (0.05/5 = 0.01) and two saturated fatty acids (0.05/6 = 0.0083) significant indices, as can be seen in Table 3. Percentages of the saturated fat lignoceric acid (24:0), and most types of trans-fatty acids (18:1t, 18:2n6ct, and 18:2n6tc) were significantly higher in children with vs. without ADHD (Table 3). The monounsaturated fat nervonic acid (24:1n9), showed a trend toward significance with higher levels in ADHD vs. control groups. The levels of Stearic acid (18:0) were significantly lower in the ADHD group, compared to children without ADHD (Table 3). The average omega-3 index of children diagnosed with ADHD was slightly lower (4.45%) compared to children without ADHD (5.06%). This difference was close to significance (*p* = 0.055, Table 3). The levels of total trans-fatty acids within the ADHD group did not differ between children receiving medication and those that do not (0.71 ± 0.18 vs. 0.61 ± 0.20, *p* = 0.126).

**Table 3:** Fatty acid profiles from RBC membranes comparing children with ADHD vs. without (control).

**Fatty acid profiles**				**ADHD** ***n*** **= 32**	**Control** ***n*** **= 28**	***p*** ^*^
**Saturated**		Myristic 14:0		0.24 ± 0.08	0.21 ± 0.05	0.084
		Palmitic acid 16:0		21.37 ± 0.63	21.39 ± 0.83	0.885
		**Stearic 18:0**	↓	17.43 ± 0.81	18.00 ± 0.79	0.008^*^
		Eicosanoic 20:0		0.15 ± 0.03	0.15 ± 0.05	0.782
		Docosanoic 22:0		0.23 ± 0.05	0.22 ± 0.09	0.551
		**Lignoceric 24:0**	↑	0.61 ± 0.15	0.50 ± 0.17	0.008^*^
**Mono saturated**		Palmitoleic 16:1n7		0.22 ± 0.08	0.19 ± 0.06	0.214
		Oleic 18:1n9		14.56 ± 1.06	14.44 ± 0.87	0.617
		Eicosenoic 20:1n9		0.29 ± 0.10	0.33 ± 0.19	0.321
		Nervonic 24:1		0.62 ± 0.13	0.53 ± 0.18	0.052
**Polyunsaturated**	**Omega-6**	**Linoleic 18:2n6**	↑	12.79 ± 1.41	11.93 ± 0.98	0.008
		gamma Linolenic 18:3n6		0.09 ± 0.02	0.09 ± 0.03	0.200
		Eicosadienoic 20:2n6		0.25 ± 0.05	0.23 ± 0.04	0.110
		**DGLA 20:3n6**	↓	1.74 ± 0.43	1.95 ± 0.39	0.049
		Arachidonic 20:4n6		17.14 ± 1.37	17.47 ± 0.99	0.174
		Docosatetraenoic 22:4n6		3.81 ± 0.64	3.54 ± 0.46	0.070
		**Docosapentaenoic 22:5n6**	↑	1.15 ± 0.23	1.01 ± 0.24	0.022
		**Total omega-6**	↑	36.78 ± 1.23	36.09 ± 1.25	0.034
	**Omega-3**	**ALA 18:3n3**	↑	0.16 ± 0.05	0.13 ± 0.03	0.021
		EPA 20:5n3		0.39 ± 0.14	0.38 ± 0.17	0.802
		Docosahexaenic 22:5n3		1.99 ± 0.29	2.03 ± 0.31	0.602
		**DHA 22:6n3**	↓	4.06 ± 0.79	4.68 ± 1.37	0.040
		Total omega-3		6.60 ± 0.87	7.22 ± 1.52	0.062
		Omega-3 Index		4.45 ± 0.86	5.06 ± 1.44	0.055
**Trans**		**16:1n7t trans**	↑	0.07 ± 0.03	0.05 ± 0.02	0.031
		**18:1t trans**	↑	0.44 ± 0.16	0.33 ± 0.14	0.005^*^
		18:2n6tt trans		0.006 ± 0.01	0.004 ± 0.01	0.606
		**18:2n6ct trans**	↑	0.014 ± 0.01	0.008 ± 0.01	0.021
		**18:2n6tc trans**	↑	0.11 ± 0.03	0.09 ± 0.03	0.006^*^
		**Total trans**	↑	0.64 ± 0.21	0.49 ± 0.18	0.003^*^

*Analysis of fatty acids from red blood cell (RBC) membranes was performed via gas chromatography. Fatty acid values are expressed as percentage of total fatty acids and reported as mean ±standard deviation. Mann-Whitney non-parametric tests was used to compare individual fatty acid percentages between ADHD and non-ADHD groups. p-value ≤ 0.05 shown in bold, points to a significant difference. Arrows represent whether mean relative abundance was higher (↑) or lower (↓) in the ADHD vs. control groups. ADHD, attention-deficit hyperactivity disorder; DGLA, dihomo-gamma-linolenic acid; ALA, alpha linolenic; omega-3 index was calculated as sum of EPA, eicosapentaenoic acid and DHA, docosahexaenoic acid*.

* *Significant after Bonferroni correction according to the subgroups (Saturated 0.0083, Omega-6 0.0071, Omega-3 0.0125, Trans 0.01). Fatty acids with p-value ≥ 0.05 shown in bold*.

We next analyzed the levels of trans-fatty acids according to CGI-S scale assessment by psychiatric examination. We found that the percentage of total trans-fats was higher in the extremely severe clinical condition score CGI-S = 6, and CGI-S = 5, as compared to control subjects.

## Discussion

Our study aimed to evaluate the association between the RBC fatty acid profile and ADHD symptomatology, as measured in a sample of Israeli children with vs. without ADHD.

We identified various alterations in the fatty acid profile of children with ADHD compared to controls. Three types of trans-fatty acids were represented by significantly higher levels in ADHD vs. control children. Additionally, the percentage of total trans-fats was higher in the extremely severe ADHD, and in milder ADHD as compared to control subjects.

Concerning the composition of saturated fatty acids, we found higher levels of lignoceric acid (24:0), whereas significantly lower levels of stearic acid (18:0) in children with ADHD. Interestingly, high levels of lignoceric acid in RBCs were previously found to be associated with inflammation in adults with metabolic syndrome (29). Decreased levels of the serum sphingomyelin 18:0 were previously found in ADHD patients (30). Regarding monounsaturated fatty acids, nervonic acid (24:1n9) showed a trend toward significance with higher levels in the ADHD group vs. controls. These findings are in contrast to previous work showing lower levels of nervonic acid in RBC membranes in children with vs. without ADHD in Taiwan (31). Interestingly, according to the literature, nervonic acid was previously suggested as a good candidate biomarker for a depressive state (30), as well as a predictor for conversion to psychosis in young people at high clinical risk for psychosis (31). However, the clinical relevance of nervonic acid to ADHD remains unknown.

The mean omega-3 percentage in healthy Israeli children was found in our study to be 5.06%, which is significantly higher than values reported for healthy children from some other countries (3.5% in children in the United States, and 4.9% in Australian adolescents), while lower than the index of 5.7% found in adolescents in Norway (32). This may be important to take into account when comparing values from studies performed on different populations around the globe, and may reflect the different diets of children at various geographical locations.

While not reaching significance, we found a lower mean omega-3 index in children with ADHD (4.45%), compared to the non-ADHD control group. We additionally found that levels of the docosahexaenoic acid (DHA) component of the omega-3 index, the main omega-3 in the CNS, were lower in the ADHD group, compared to the control group without ADHD. However, Eicosapentaenoic acid levels did not differ significantly between groups.

In relation to the omega-6 family, levels of linoleic acid and docosapentaenoic acid were higher in the ADHD group vs. controls. The only omega-6 that was lower in children with ADHD vs. controls was dihomo-gamma-linolenic acid (DGLA), matching results of a previous study among hyperactive children (33). Additionally, lower DHA and DGLA levels, as well as higher linoleic acid levels, were previously found in ADHD adolescents when compared to age-matched control subjects (9). Dihomo-gamma-linolenic acid is an omega-6, which can be further converted into anti-inflammatory prostaglandin. It has been suggested that DHA and DGLA and their metabolites have pro-immune actions in addition to their prominent role in the brain (34).

Although values of BMI were within the normal range in both study groups, it is important to note that a significant difference in BMI was observed in children with vs. without ADHD, with a higher average within the ADHD group. This result has also been found by others (35), suggested that the etiological factors influencing the association between ADHD and BMI may differ by demographic characteristics (36). Mechanistically, there may be a connection between the higher trans-fatty acid levels we found in the ADHD group and the higher BMI levels, but no significant correlations found here. It has been shown in monkeys that long-term trans-fatty acid consumption is an independent factor in weight gain (37). A rat study which aimed to test the effects of trans-fatty acids on markers of obesity and pre-diabetic state suggested that trans-fatty acids altered nutrient handling in liver, adipose tissue, and skeletal muscle as well as the mechanism by which trans-fatty acids induce insulin resistance (38). There have also been some epidemiological studies showing the same trends (39). However, the precise potential mechanisms linking between ADHD, trans-fatty acids, and higher BMI require further investigation (40). For instance, it is of particular interest whether the dietary consumption levels of trans-fatty acids differ between children with vs. without ADHD.

In this study, we chose measurements of blood trans-fatty acid levels, as these are generally more reliable and comprehensive than self-reported intake assessment, which are limited in the ability to accurately reflect the nutrient consumption of individuals due to measurement error, recall bias, selective report, and incompleteness of food composition databases (41). If indeed the relatively high levels of trans-fatty acids observed in RBCs also reflect high levels in brain cell membranes, they may perhaps take part in the pathophysiology of ADHD. The physiological implication could be due to trans-fatty acids interfering with the desaturation and elongation of both omega-6 and omega-3 fatty acids, thus further decreasing their availability for metabolism. Another possibility is that since trans-molecules are more static, they may reduce the flexibility of the membranes. The mechanism by which trans-fatty acid isomers influence health outcomes remains elusive (42). One suggestion is that the toxicity of trans-fatty acids may reside in their ability to inhibit cytoprotective stress responses induced by saturated fatty acids (43). Our study is unique as only few studies on trans-fatty acids in children have been performed previously. Despite the correlation found between fatty acid profiles and ADHD status, it remains unknown whether increased trans-fatty acid levels can cause or exacerbate ADHD, or whether higher trans-fatty acid levels in blood are a result of a contributory mechanism. Higher levels of trans-fatty acids may be a result either of higher dietary consumption or altered metabolism of fatty acids.

In general, our results present a unique RBC fatty acid profile for children with vs. without ADHD, highlighting observed differences in specific fatty acids. Due to the numerous differences, it remains difficult to assess the precise clinical effect of each fatty acid separately by this analysis. Additionally, our study is observational, therefore it cannot provide causal evidence of an effect of trans-fatty acids on the development of health outcomes examined; it can only describe associations. However, certain observations, such as the relatively high levels of trans-fatty acids in children with ADHD present potential directions for future research and potential interventions.

## Conclusions

Our study reveals significant differences in the composition of RBC fatty acids in children with vs. without ADHD. Higher relative levels of most trans-fatty acids were found in children with ADHD vs. control. Future studies should be designed to evaluate the clinical relevance of trans-fatty acids in a larger cohort of ADHD subjects, together with dietary analysis, and perhaps evaluate elimination of trans-fats as a nutritional intervention.

## Data Availability Statement

The raw data supporting the conclusions of this article will be made available by the authors, without undue reservation.

## Ethics Statement

The studies involving human participants were reviewed and approved by Ziv Helsinki Committee, Israel Ministry of Health. Written informed consent to participate in this study was provided by the participants' legal guardian/next of kin. Written informed consent was obtained from the minor(s)' legal guardian/next of kin for the publication of any potentially identifiable images or data included in this article.

## Author Contributions

AA-O, EA, ST, and UY: designed research. AA-O, EA, KS, IM, MS, and UY: conducted research. AA-O and HN: analyzed data and wrote the paper. All authors read and approved the final manuscript.

## Conflict of Interest

The authors declare that the research was conducted in the absence of any commercial or financial relationships that could be construed as a potential conflict of interest.

## Publisher's Note

All claims expressed in this article are solely those of the authors and do not necessarily represent those of their affiliated organizations, or those of the publisher, the editors and the reviewers. Any product that may be evaluated in this article, or claim that may be made by its manufacturer, is not guaranteed or endorsed by the publisher.
